# The Treatment of PPCP-Containing Sewage in an Anoxic/Aerobic Reactor Coupled with a Novel Design of Solid Plain Graphite-Plates Microbial Fuel Cell

**DOI:** 10.1155/2014/765652

**Published:** 2014-08-14

**Authors:** Yi-Tang Chang, Chu-Wen Yang, Yu-Jie Chang, Ting-Chieh Chang, Da-Jiun Wei

**Affiliations:** ^1^Department of Microbiology, Soochow University, 70 Linhsi Road, Shilin District, Taipei 11102, Taiwan; ^2^Department of Earth and Life Science, University of Taipei, Taipei 11048, Taiwan

## Abstract

Synthetic sewage containing high concentrations of pharmaceuticals and personal care products (PPCPs, mg/L level) was treated using an anoxic/aerobic (A/O) reactor coupled with a microbial fuel cell (MFC) at hydraulic retention time (HRT) of 8 h. A novel design of solid plain graphite plates (SPGRPs) was used for the high surface area biodegradation of the PPCP-containing sewage and for the generation of electricity. The average COD_Cr_ and total nitrogen removal efficiencies achieved were 97.20% and 83.75%, respectively. High removal efficiencies of pharmaceuticals, including acetaminophen, ibuprofen, and sulfamethoxazole, were also obtained and ranged from 98.21% to 99.89%. A maximum power density of 532.61 mW/cm^2^ and a maximum coulombic efficiency of 25.20% were measured for the SPGRP MFC at the anode. Distinct differences in the bacterial community were presented at various locations including the mixed liquor suspended solids and biofilms. The bacterial groups involved in PPCP biodegradation were identified as *Dechloromonas* spp., *Sphingomonas* sp., and *Pseudomonas aeruginosa*. This design, which couples an A/O reactor with a novel design of SPGRP MFC, allows the simultaneous removal of PPCPs and successful electricity production.

## 1. Introduction

Pharmaceuticals and personal care products (PPCPs) are being paid more public attention as emerging organic contaminants (EOCs) in ecosystems. In Taiwan, the existence of pharmaceuticals pollution can be attributed to incomplete municipal sewage collection systems and inappropriate recycling and treatment programs for waste medical materials. Pharmaceutical sewage can be treated by municipal wastewater treatment plants (WWTPs), but the effluent obtained by such plants introduces residues into the hydrological cycle [1]. Natural surface water systems, such as rivers, reservoirs, and oceans, are widely distributed in Taiwan and are, thus, prone to contamination. Similarly, urban groundwater reservoirs are likely to be contaminated by pharmaceuticals from sewage. The concentration of such pharmaceuticals has been found to range from ng/L to *μ*g/L and can even reach mg/L levels in sanitary landfill leachates. Typical examples of the pharmaceutical drugs found in such sewage in Taiwan include analgesics, antibiotics, and nonsteroidal anti-inflammatory drugs (NSAID); examples are acetaminophen (ACE), sulfamethoxazole (SMX), and ibuprofen (IBU); such compounds are often detected at relatively high concentrations (*μ*g/L) in the influent of municipal WWTPs [2].

Anoxic-aerobic coupled systems (A/O systems) have been applied to the treatment of a wide range of municipal wastewaters and industrial wastewaters of low-to-high strength concentrations. The advantages of A/O systems include a high overall treatment efficiency with respect to BOD and nutrients, a reduced need for sludge disposal, a lower consumption of chemicals, and a greater potential for resource recovery [3]. The combination of aerobic and anoxic degradation pathways in the A/O system has been reported as aiding the overall degradation efficiency of PPCPs. The removal efficiencies for PPCPs in municipal wastewater vary on a case-by-case basis due to differences in the biological processes and the target PPCPs. For example, biofiltration of wastewater through biological activated carbon has shown good potential for the efficient removal of PPCPs (>90%), but sand biofilters have a poor efficiency [4]. Sulfonamides have been shown to be removed in the range from 18.5% to 37.2% using an anoxic/anaerobic/aerobic (A2O) system, but higher removal efficiencies from 53.3% to 73.3% were obtained using an oxidation ditch [5]. Thus, it is necessary to clarify the contribution to sewage treatment efficiency of each different reactor in an A/O system.

Energy is required to keep the regular operations at an A/O system fully powered, for example, to provide oxygen (O_2_) to the aerobic reactor. Recently, microbial fuel cells (MFCs) have been found to be able to provide an innovative renewable energy source that is both green and clean, as well as having a low carbon footprint [6, 7]. The potential for electrical generation of MFCs is being developed and this approach as a source of green energy has the potential to reduce various operational costs (electricity) including aeration and recirculation/process pumping. However, when applied, the two-chamber MFC A/O system is limited in terms of the amount of electrode design. Alternative approaches that can be implemented include improving the electrode design and/or modifying the system by using various chemical catalysts. For example, modifying the cathode by adding a noble metal such as Pt or a nonnoble metal such as Fe^3+^ or Mn^4+^, which can then act as a chemical mediator, is able to significantly increase the PD of MFCs [8, 9]. Such advances in electricity production either need specific carbon-based materials or have to include noble metals at extremely high cost [10], both of which retard the practical development of such systems for MFCs that are coupled with the biological treatment of wastewater. In addition, some of the above mediators are known to be toxic to the growth of bacteria, which are central to biodegradation.

Graphite electrodes in a MFC system are good in terms of power production compared to various metals, such as iron, aluminum, and stainless steel [11]. Biofilms on the graphite electrodes are known to contribute to power production in the MFC system. Different types of graphite cathode/anode electrodes, including graphite plates, sheets, felts, rods, and papers, have been developed to increase electricity output [12]. In fact, the bacterial biofilm formed on the graphite material is also able to biodegrade sewage, even EOCs. The development of high specific surface area graphite cathode/anode electrodes should result in great benefits in a MFC A/O system in terms of generating electricity and sewage removal. Moreover, the bacterial community structures and their functions are complex in a MFC A/O system. Till now, there has been little information available on the influence of PPCP-containing sewage on the various dynamic bacterial communities present in A/O systems and on electricity generation during biological treatment by an A/O system.

The objective of this study is to design and implement a MFC system that is combined with an A/O reactor and to use this system to process PPCP-containing sewage for the first time. MFC solid plain graphitic plates (SPGRPs) were designed to generate bacterial electricity and to remove PPCPs in a highly effective manner. The removal efficiencies, including COD, N, and P, when treating the sewage, were measured using an integrated MFC A/O system. At the same time, PPCP biodegradation was assessed. The spatial bacterial communities and their major functions were carefully evaluated using biomolecular methods, namely, PCR-DGGE-cloning. The biodiversity of the bacterial communities at various locations, such as mixed liquor suspended solids (MLSS) and biofilms, was compared across the MFC A/O system. This study provides an alternative approach to the biological treatment of municipal/industrial wastewater that contains PPCPs; specifically, it involves the coupling of a two-chamber MFC to an A/O reactor. The specific functions of the various members of the bacterial population present in the reactors were clarified in terms of a series of biochemical reactions within the MFC A/O system.

## 2. Material and Methods

### 2.1. Chemicals

Three target pharmaceutical drugs among potential PPCPs were selected for this study, ACE, SMX, and IBU. These drugs are commonly found in WWTP municipal wastewater in Taiwan. ACE was purchased from Fluka at purity of >98%. SMX and IBU were obtained from Sigma-Aldrich at purity of >99% and Sigma at purity of >99.9%, respectively. The organic solvents used in this study were all HPLC grade with purity higher than 99.9%. All other chemicals were reagent grade with purity above 99%. The Milli-Q water was double-distilled and deionized by a Millipore water purification system.

### 2.2. The MFC A/O System

**Figure 1:**
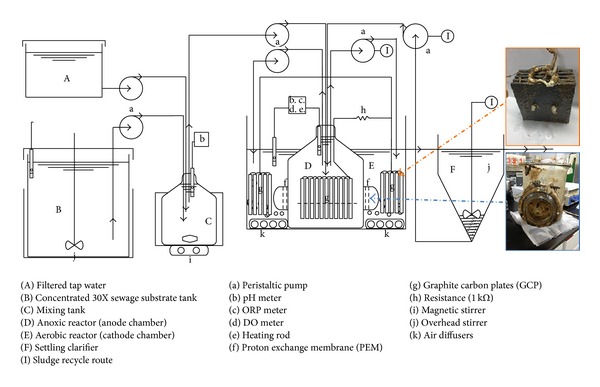
Schematic diagram of the A/O reactor and the MFC coupled system.

Figure 1 shows the pilot-scale coupled MFC A/O system used in this study. The sewage influent consisted of a mixture of condensed artificial PPCP-containing sewage and tap water in a stabilized tank (25,000 mL) that was controlled to a temperature between 8°C and 12°C. The anoxic reactor (3,940 mL) was designed as the inner tank and its temperature was controlled to be within the range from 26°C to 29°C, while the aerobic reactor (11,565 mL) was designed as the outer tank. The MFC system consisted of the inner tank (cathode chamber) and outer tank (the anode chamber) separated by two proton exchange membranes (PEM, Nafion N117, DuPont Nafion PFSA membrane). The total area of PEM in the MFC A/O system was 68.40 cm^2^ and had the ability to transport hydrogen as protons from the anode (anoxic tank) to the cathode (aerobic tank). SPGRPs (96 mm × 36 mm × 5 mm) with high specific surface areas (20,267.22 mm^2^ for each SPGRP) were used for two different purposes in this study, with one set being in the cathode chamber and another set being in the anode chamber. The SPGRPs were fixed by two PTFE-covered stainless steel bars. Copper wires were used to connect all the SPGRPs within the MFC system. The anoxic reactor included eleven SPGRPs that were designed to allow the development of bacterial biofilms on the cathode (called the cathode catalysts or the biocathode) that would increase electricity generation by the MFC. In contrast, the anode chamber consisted of ten SPGRPs that were designed to allow the formation of biofilms that would aid the removal of artificial PPCPs from the sewage.

### 2.3. Inoculation and Experimental Operation of the MFC A/O System

The original source of the active sludge used to inoculate the pilot-scale coupled MFC A/O system came from a secondary sedimentation tank at the Neihu WWTP, Taipei, Taiwan, which is used to treat PPCP-contained sewage. To avoid the influence of the complex content found in real sewage during PPCP biodegradation, artificial sewage containing the target PPCPs was used in this study.  Table 1 presents the components present in the artificial PPCP-containing sewage used in this study. The COD/N/P ratio of influent artificial sewage is about 257.16 : 13 : 1.96, which is quite close to the best composition (C/N/P = 100 : 5 : 1) for municipal wastewater when carrying out biological treatment at a WWTP. Activated sludge in the settlement tank (154,000 mL) was set up to be 100% recycled into the anoxic reactor because increasing the sludge retention time will reduce the operation costs. Water parameters, including pH, ORP, and DO, were obtained by real-time monitoring of all tanks.  Table 2 lists the operating parameters of the MFC A/O system. In order to confirm the ability of the system to remove PPCPs and to be able to observe the shifts in bacterial community present in the MFC A/O system, the influent concentrations of ACE, SMX, and IBU were designated to be 30, 2, and 20 mg/L, respectively, which are about 1,000-fold higher concentrations than those present in sewage effluent from Taiwan. The continuous flow rate of the sewage influent was controlled to be 32.33 mL/min during this experiment. The hydraulic retention time (HRT) was set up to be 8 h, which consisted of 2.04 h in the anoxic reactor and 5.96 h in the aerobic reactor. Two experimental phases were carried out as part of this study. Phase I was designed to have the A/O system coupled with MFC system in steady operation for the biological treatment of artificial sewage without PPCPs and this lasted 95 days. Phase II involved treatment of PPCP-containing sewage and took place immediately after Phase I; this phase lasted for 28 days. The treatment of the PPCPs, the water parameters (COD_Cr_, N, and P), and the bacterial community present were examined regularly.

### 2.4. Electricity Measurements and Calculations

Power density (PD) and coulombic efficiency (*E*_*C*_) were selected to be evaluated as measures of the electricity generation by the MFC system [13]. Voltage (*V*) was regularly measured using a multimeter (LTlutron DM-9090, Taiwan) via a data acquisition system and this was converted to PD. PD is the power (*P*: the definition is the time rate of energy transfer) per cross-sectional area (projected) of the anode (*A*) according to following equations:

(1) $$\begin{array}{c} I\left(\text{mA}\right)=\frac{V}{R},\\ P\left(\text{mW}\right)=I\times V,\\ \text{PD}\left(\text{mW}/{\text{m}}^{2}\right)=\frac{P}{A}, \end{array}$$

where *P* is the power, *I* is the current (mA), and *R* is the resistance.

The *E*_*C*_ is calculated based on the ratio of total electrons recovered as *I* to maximum possible electrons recoverable if all substrate removal produces current; this is calculated using the following equation:

(2) $$\begin{array}{c} E_{C}=\frac{C_{P}}{C_{\max}}\times 100\%, \end{array}$$

where *C*_*P*_ is the total coulombs calculated by integrating the current over time. *C*_max⁡_ is the theoretical amount of coulombs that can be produced from the artificial wastewater, calculated using the following equations:

(3) $$\begin{array}{c} C_{P}=I\times \text{HRT},\\ C_{\max}=FfS_{\text{COD}}V_{\text{anode}}, \end{array}$$

where HRT is hydraulic retention time in the MFC A/O system (s); *F* is Faraday's constant (96,485 C/mol of electrons); *f* is the number of moles of electrons produced per mole of sewage (1/8 mol of electrons/g COD); *S*_COD_ is the difference in COD between the influent and effluent in the anode chamber (anoxic reactor); *V*_anode_ is the effective volume of anode volume.

### 2.5. Water Parameters Analysis

Samples of artificial PPCP-containing sewage were initially passed through a 1.20 *μ*m glass-fiber membrane and then refiltered through a 0.45 *μ*m nylon membrane. Samples for water parameter analysis were acquired from the same reactor and at the same time as the microbial samples. Water parameters, including temperature, pH, SS, VSS, and COD_Cr_, were analyzed and this was done by following the procedures from the Standard Methods for the Examination of Water and Wastewater [14]. Total nitrogen (T-N) and total phosphate (T-P) were measured using test kits, namely, Merck spectroquant Nova 60. NH_4_^+^–N, NO_2_^−^–N, NO_3_^−^–N, and PO_4_^3−^–P were measured by ion chromatography (IC, Metrohm 883 Basic IC, USA). Real-time pH/ORP and DO were monitored using a pH/ORP meter (LTlutron pH/ORP-208 meter, Taiwan) and a DO meter (EZDO, PDO-408, Taiwan), respectively.

### 2.6. PPCPs Analysis

Filtered sewage samples are dried into a powder on a freeze vacuum evaporator (Labconco, USA) at −50°C. Extracted samples were concentrated by hexane and diluted using acetonitrile (ACN) to adjust the concentration correctly. The stock solution of PPCPs for the HPLC standards was prepared by serial dilution in ACN and stored in dark-brown glass containers at 4°C to prevent photolysis of the PPCPs. Samples and standards were injected into the HPLC system to determine the concentration of PPCPs. The HPLC system was equipped with a UV detector (YL-9100, Young-Lin, Korea) and C18 column (250 × 4.6 mm, Thermo Scientific, USA). The operating conditions of HPLC were as follows: 15 *µ*L injection sample and 1.2 mL/min mobile phase composed grade ACN and 0.02 M phosphoric acid (PA) in the gradient program. The recovery range for the PPCPs in samples was from 75% to 95% and the losses were probably due to limitations of the analytical methods. The detection limit of this approach (MDL) to the analysis of the target PPCPs was 5 *μ*g/L. Triplicate analyses of the PPCPs were carried out on each sample.

### 2.7. Bacterial Community

#### 2.7.1. DGGE

The genomic DNA of microorganisms involved in the A/O system was extracted from MLSS, SPGRP biofilms, and PEM biofilms in the MFC A/O system using a soil genomic DNA purification kit (Gene Mark, Taiwan). Bacterial 16S rDNA genes were selectively amplified from the purified DNA products by PCR. The V6–V8 region of 16S rDNA was selected using the forward primer 968F-GC clamp and the reverse primer 1392R [15]. The DNA product was separated by DGGE profiling using DCode Universal Mutation Detection System (Bio-Rad Laboratories, Inc., Hercules, California, USA) and 40% to 65% gradient gel at 60°C and 110 V for 16 h. The acrylamide percentage used for the DGGE electrophoresis gel was 8% and the denaturing agents were formamide and urea. Richness indices (RIs), which are related to the band numbers on the DGGE profiles, were used to represent the variation in biodiversity of the MFC A/O system. This allows the assessment of the changes in richness of the bacterial populations.

#### 2.7.2. Cloning

The genomic DNA of microorganisms involved in the A/O system was extracted from MLSS, GRP biofilms, and PEM biofilms in the MFC A/O system using a soil genomic DNA purification kit (Gene Mark, Taiwan). Bacterial 16S rDNA genes were selectively amplified from the purified DNA products by PCR. Clone libraries were then constructed after amplifying the full length (including the V1–V8 region) of the 16S rRNA using the forward primer E9F and the reverse primer U1510R [16]. The amplicons were purified using an EasyPure PCR/Gel Extraction kit (Bioman, Taiwan). The clean product was then cloned using the pGEM-T Easy Vector Systems kit (Promega, Madison, Wisconsin, USA) and transformed into competent* Escherichia coli* DH5a cells as described by the manufacturer. The transformed* E. coli* was incubated on LB agar plates at 37°C overnight and the next day the blue-white screening method was applied to select all white colonies from each population. Plasmids DNA from each colony was then extracted using an EasyPure Plasmid DNA miniprep kit (Bioman, Taiwan). Plasmids with the correct DNA insert were identified by the PCR amplification using the primers M13-F (5′-GTT-TTC-CCA-GTC-ACG-AC-3′) and M13-R (5′-ACA-GGA-AAC-AGC-TAT-GA-3′). The DNA sequencing of the various 16S rRNA inserts was carried out by the Genomics Company, Taiwan. All sequences were compared with reference microorganisms from the GenBank database using BLAST. The closest 16S rDNA sequences to the 16S rRNA sequences obtained from the bacteria making up the biodegradation bacterial populations were retrieved and all the sequences were then aligned using Clustal X software. A phylogenetic tree was constructed by the neighbor-joining method using Molecular Evolutionary Genetics Analysis, version 5 (MEGA 5.1 Beta 3) software. Bootstrap values of >1,500 (from 5,000 replicates) are indicated as at the nodes in the phylogenic analysis.

## 3. Results

### 3.1. Treatment of PPCP-Contained Sewage

**Figure 2:**
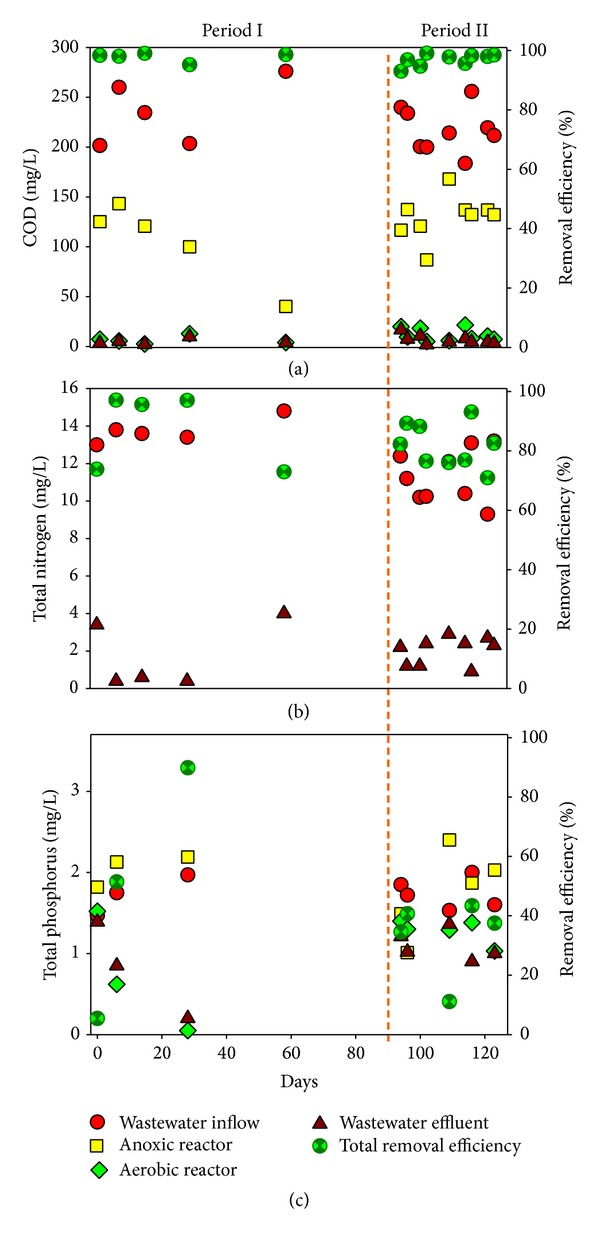
Variation in water parameters in the MFC A/O reactor: (a) COD_Cr_; (b) T-N; (c) T-P. Phase I = 95 days; Phase II = 28 days.

Figure 2 outlines the variation in water parameters of the MFC A/O system during Phases I and II (totally 125 days). There is no significant difference in sewage removal when Phase I and Phase II are compared (ANOVA), which indicate that the performance of biological treatment is not affected by the presence of PPCPs. The total removal efficiency of the COD_Cr_ averaged 97.20%. The contributions of the anoxic reactor and aerobic reactor to COD_Cr_ removal were 44.80% and 50.61%, respectively. The total removal of T-N averaged 83.75% for the complete A/O system. In contrast, the total removal of T-P averaged only 39.24%, but this was because the sludge settlement in secondary settlement tank was not disposed of on a regular basis. The present MFC A/O system showed a better biological treatment performance compared to a previous study where the removal efficiency for COD_Cr_, T-N, and T-P during the biological treatment of sewage containing 20 PPCPs by a WWTP at 8 h HRT was found to be 75.0%, 42%, and 66.0%, respectively [17].

Table 3 shows the average concentrations of specific nutrients that were present in the high strength PPCP-containing sewage of the MFC A/O system over Phases I and II. The SPGRP biofilms within the MFC provided simultaneous nitrification and denitrification in the study. Basically, biofilms on the SPGRP bring about denitrification in the anoxic reactor, while the SPGRP biofilms allow parallel nitrification and aerobic oxidation in the aerobic reactor. The membrane of the PEM contains sulfonic acid groups, which are able to bind the ammonia present during the aerobic nitrification. The concentration of NH_4_^+^–N in effluent was reduced from 1.767 ± 0.894 mg/L to 0.036 ± 0.009 mg/L in effluent by nitrification/denitrification through the complete A/O reactor process. The total removal efficiency for NH_4_^+^–N was 97.96%. A significantly increased concentration of NH_4_^+^–N was found in the anoxic reactor of 9.02 ± 3.62 mg/L because of the mixing of sewage influent and 100% recycled settlement sludge. The concentrations of nitrite and nitrate were found to be decreased in the anoxic reactor. A removal efficiency of 83.28% for nitrate was measured with a biological reduction from 1.555 ± 0.501 mg/L to 0.260 ± 0.076 mg/L. Nitrification was found to occur in aerobic reactor, where the concentration of nitrate was increased from 0.260 mg/L to 1.033 mg/L. Since the A/O process is not designed as a T-P removal system, the low removal efficiency observed is not unexpected. The concentration of PO_4_^3−^–P was slightly decreased from 1.31 ± 0.29 mg/L to 1.090 ± 0.422 mg/L. Moreover, the concentration of T-P averaged 1.8675 ± 0.4412 mg/L in anoxic reactor, which is greater than that of the aerobic reactor at 1.0738 ± 0.500 mg/L. This can be ascribed to intracellular polyphosphate (poly-P) being taken up into the biomass present in the aerobic reactor and then being released in the anoxic reactor. However, the target PPCPs in the present system might have had an effect on nutrient removal in the A/O MFC system. For example, 50–500 mg/L of IBU and ACE have been shown to inhibit nitritation/denitritation and phosphorus uptake/release rates in a sequence of batch reactors [18].

### 3.2. Occurrence and Removal of PPCPs

**Figure 3:**
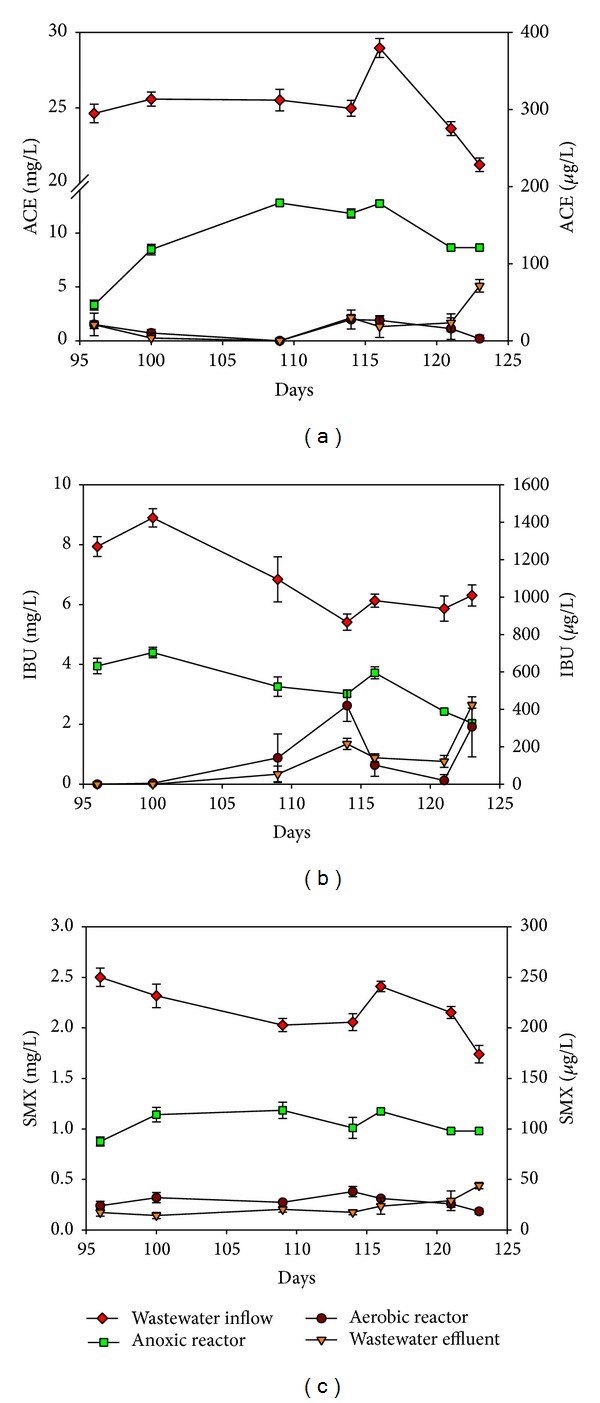
Variation in targeted PPCPs in the MFC A/O system during Phase II: (a) ACE; (b) IBU; (c) SMX. The concentrations in the sewage influent (◆) and in the anoxic reactor (■) are presented on the left-*Y* axial (mg/L). The concentrations in the aerobic reactor (▼) and in the sewage effluent (●) are presented on the right-*Y* axial (*μ*g/L).

Figure 3 shows the variation of PPCP concentration throughout the MFC A/O system. High concentration PPCPs (mg/L level) in the artificial sewage were removed at an efficiency greater than 98% in this MFC A/O system. The PPCP removal performances were compared and this gave the following result (ANOVA, *P* < 0.05): ACE (99.89%) > IBU (99.01%) > SMX (98.21%). A similar trend in terms of removal efficiencies of 99.8-99.9% for ACE, 99.1–99.5% for IBU, and 73.8–80.8% for SMX was found using a conventional activated sludge WWTP system linked to two pilot-scale membrane bioreactor treatment (MBR) systems [19]. In general, antibiotics such as SMX are more resistant to biodegradation in most WWTPs than other pharmaceuticals. It has been reported that 10–400 mg/L SMX is able to inhibit microbial activity in activated sludge by more than 20% [20]. In one study, an average removal efficiency of 65% for SMX was achieved by MBR under anoxic and aerobic conditions [21].

PPCPs at a ppb level could still be detected by HPLC in the effluent of the conventional A/O process. The ACE, SMX, and IBU effluent concentrations were 23.9 ± 2.34 *μ*g/L, 23.7 ± 1.1 *μ*g/L, and 179.9 ± 17.7 *μ*g/L, respectively, and these levels still might pose an ecological risk in terms of the aquatic environment. Since groundwater constitutes the main source of public drinking water supplies in many countries, people who drink PPCPs-contaminated water may suffer an adverse effect on their growth and reproduction. Specific pharmaceuticals at low concentrations (ng/L) have become an important issue, particularly because of their toxicity towards living organisms. For example, about 50% to 90% of the original SMX dose and its metabolites are released into the environment and these then bioaccumulate via biotic factor and abiotic factors in the food chain [22]. SMX induces antibiotic resistance in bacteria and hazard quotients in WWTP effluent have revealed that these chemicals may pose an ecotoxicological risk to algae [23]. The occurrence of ACE has been reported in the aquatic environment and there is an important need to address the potential toxic effects of ACE on nontarget environmentally exposed organisms [24]. Exposure to low concentrations (10–100 ng/L) of IBU has been found to result in a significant decrease in the activity of* Gammarus pulex* [25].

### 3.3. Electricity Production by the MFC A/O System

**Figure 4:**
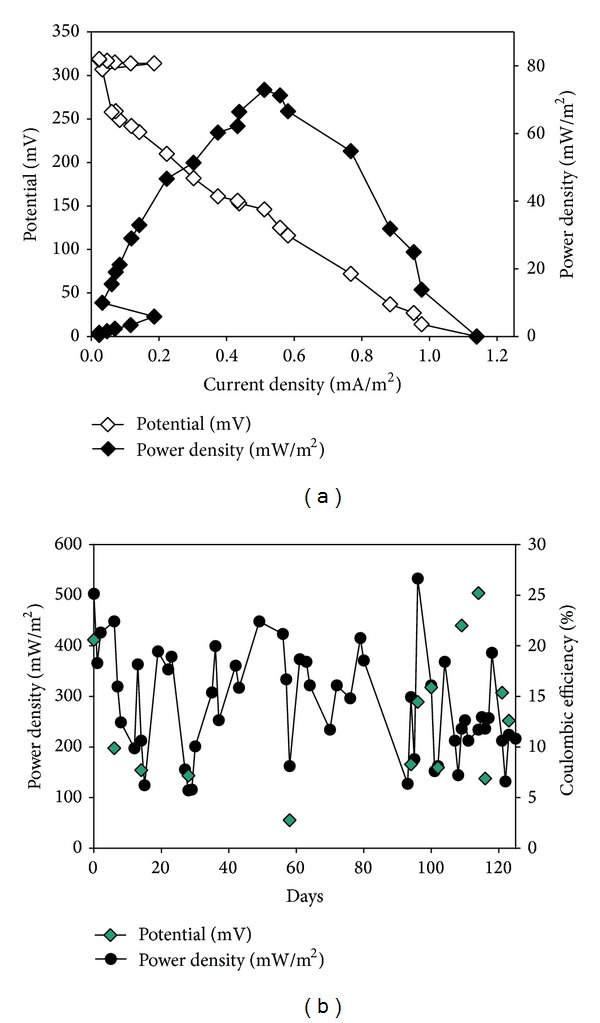
Electrical generation by the A/O reactor coupled with the novel MFC system: (a) polarization curve; (b) power density and coulombic efficiency.

Figure 4 demonstrates electricity generation by the MFC A/O system. Initially, polarization curves were obtained by measuring the power generation at various external resistances (from 510 KΩ to 1 Ω) and are shown in Figure 4(a). We selected 1 KΩ to measure I and A during this study. The existence of PPCPs in sewage does not seem to have affected the electric generation (ANOVA).  Figure 4(b) presents the average PD, which was found to be 285.15 mW/m^2^; furthermore, the maximum PD value achieved during Phase II was 532.61 mW/m^2^. The *E*_*C*_ values ranged widely from 2.77% to 25.20% over the 125 days of the study and averaged 12.62% overall. Direct electron transfer from microbial cells to electrodes occurs at very low efficiency and a higher PD by a SPGRP MFC. It modifies the material used as the cathode catalyst in order to increase the efficiency of the oxidation-reduction reaction. In this study, the novel design used here allows the formation of biofilms on the SPGRP, which plays an important role in the generation of electricity. Biofilms were observed to cover a high specific surface area on the SPGRPs forming both the cathode and the anode. Some aerobic bacteria might possibly be acting as cathode catalysts. The performance of a MFC has been found to increase as the biofilm develops on the cathode [26] and a high PD has been found when there is a biofilm covering the anode. This might be because the production of various biointermediates may favor electricity generation. Bacteria are able to use their respiratory chain as part of the oxidative metabolism that occurs at the anode. Nitrite might be converted to nitrate when the cathodic electrode acts as the electron donor due to denitrification in the MFC.

The PD value is higher than that in previous studies using two-chambered MFCs that have had chemical mediators added. For example, an anaerobic-aerobic sequential reactor was reported to generate 387 mW/m^2^ PD and 5.2% *C*_*E*_ with 86.4% removal efficiency when high strength dye wastewater was used that comprised 1,000 mg L^−1^ glucose and 200 mg/L Congo red (chemical mediator); this was at a longer HRT of 14.8 h [27]. A MFC shows 91% removal efficiency of high-loading domestic wastewater with the volatile fatty acid/hydrogen production which contained concentrated particular artificial food waste. The overall aim of converting chemical energy into electrical energy was achieved with a *C*_*E*_ of 46% generating 65.33 mA/m^2^ at a specific cell potential of 148 mV [28]. However, other factors can affect the generation of electricity in the MFC A/O system. The characteristics of the wastewater can affect the electrical generation performance of MFCs. The slow biodegradations of the PPCPs present in the sewage used in this study might result in more efficient electricity production. Another possible reason is that the mass transfer of protons remains a major constraint affecting the *C*_*E*_ of a MFC. The low *C*_*E*_ values are due to the fact that hydrogen proton exchange through the PEM is retarded by bacterial fouling of the A/O system. It is possible that the high internal electric resistance of the novel design for a MFC system described here might decrease electricity generation performance. Nevertheless, the dual chamber MFC A/O system still is competitive if we are considering the biological treatment efficiency of PPCP sewage and the generation of electricity at the same time.

### 3.4. The Presence of Specific Bacterial Communities in the MFC A/O System

**Figure 5:**
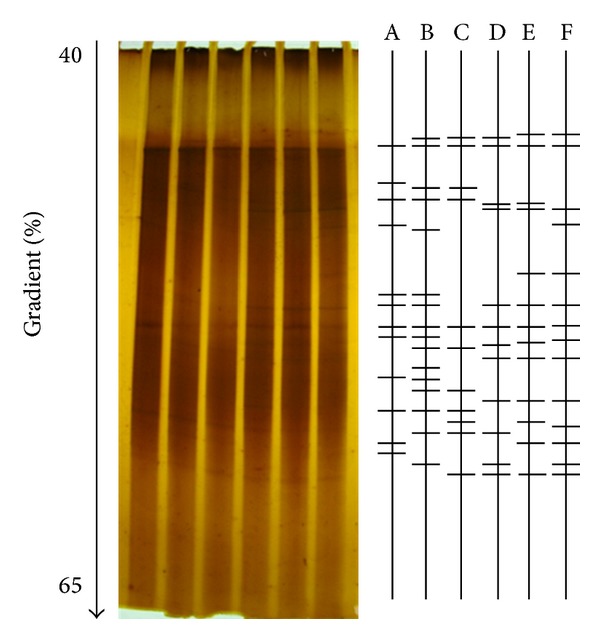
DGGE profiles analysis of the MFC A/O reactor in the MFC A/O system on the 109th day. Lines A, B, and C present the profiles of the MLSS, SPGRP biofilm, and PEM biofilm from the aerobic tank; lines D, E, and F present the profiles of the MLSS, SPGRP biofilm, and PEM biofilm from the anoxic reactor.

Figure 5 displays the biodiversity of bacterial community in the MFC A/O systems by comparing their DGGE profiles.  Table 4 compares the RI values for the DGGE bands detected across the different bacterial populations. Distinct differences were found in the bacterial species present at the three sampling locations within the MFC A/O system. The highest difference in band number ratios was 86.96% and this occurred between the SPGRP biofilms in the anoxic reactor and the PEM biofilms in the aerobic reactor. Even the lowest difference in band number ratios was as high as 65.00%, which was between the PEM biofilms in anoxic reactor and MLSS in aerobic reactor. These findings indicate the various different bacterial communities are likely to play distinctly different roles in the two chambers. For example, redox shuttling within the MFC anoxic chamber appears mainly to be present within the SPGRP and PEM biofilms and does not seem to occur within the MLSS biofilm.

**Figure 6:**
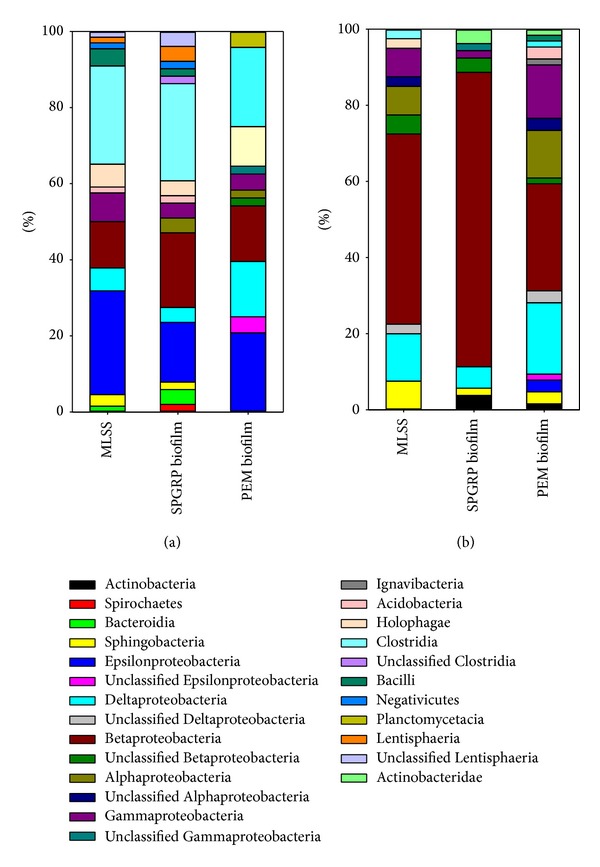
Bacterial community analysis of the MFC A/O reactor. The ratio of identified bacterial species to all bacteria cloned on the 114th day (class level): (a) anoxic reactor; (b) aerobic reactor.

Figure 6 provides detailed information on the various bacterial communities in the MFC A/O system at the class-level species using the 16S rDNA clone library. The dominant bacteria in the aerobic reactor were Proteobacteria, including *β*-Proteobacteria (53.50%), *δ*-Proteobacteria (14.65%), *γ*-Proteobacteria (8.92%), and *α*-Proteobacteria (8.92%). In addition, in terms of the three sampling locations within the reactor, the dominant species at the phylum-level are different. The percentage of *β*-Proteobacteria was 81.13% within the GRP biofilms, compared to 55.0% within the MLSS and 29.69% within the PEM biofilms. The relative abundance of *β*-Proteobacteria is probably due to the fact that several groups of aerobic or facultative bacteria are well equipped to carry out aromatic degradation. In contrast, higher percentages of *α*-Proteobacteria, *γ*-Proteobacteria, *δ*-Proteobacteria, and Sphingobacteria were found to be present in the MLSS and PEM biofilms, but these groups were found to be much less abundant in the GRP biofilm. Furthermore, the anoxic reactor within the A/O system was found to have a specific dominant bacterial community that included Clostridia-Firmicutes (24.85%), *ε*-Proteobacteria (23.03%), *β*-Proteobacteria (15.76%), Bacteroidetes (10.16%), and *δ*-Proteobacteria (7.88%). Many of these phyla can act as anode-respiring bacteria, which are defined as a bacterial population with a respiration process that can use an anode as their electron acceptor [13]. The percentage of *ε*-Proteobacteria in the MLSS was 27.27% and in the PEM biofilms was 25.0%, which should be compared with that in the GRP biofilms, which was 15.68%. Clostridia and *β*-Proteobacteria were dominant in this reactor with ranges from 20.83% to 27.45% and from 12.12% to 19.61% for the three different samples, respectively. Bacterial diversity has been found to vary at the anode of the MFCs when various different substrates are fed. For example, a two-chambered MFC using chocolate industry wastewater as the substrate had the following phyla at the anode: *α*-Proteobacteria (9.1%), *β*-Proteobacteria (50.6%), *γ*-Proteobacteria (0.8%), and Firmicutes (4.9%) [29].

## 4. Discussion

### 4.1. Comparison of PPCP Removal in the Anoxic and Anaerobic Reactors

The contribution to PPCP treatment of the aerobic reactor and of the anoxic reactor was found to be different in this MFC A/O system. The removal efficiency in the anoxic reactor averaged 62.51% for ACE, 51.88% for IBU, and 51.13% for SMX, but there was lower removal efficiency for ACE, IBU, and SMX in the aerobic reactor at 37.86%, 47.14, and 46.84%, respectively. Their biointermediates in the anoxic reactor consist of at least three known compounds (data not shown). Aerobic biodegradation has generally been demonstrated to give a better removal efficiency of COD_Cr_, which includes most of PPCPs. For example, 50 *μ*g/L of ACE and IBU were biotransformed by greater than 80% after 10 days under aerobic batch biodegradation [30]. The removal efficiency of IBU reached 95 ± 4% in an aerobic nitrification reactor but was only 37 ± 26% in an anoxic denitrification reactor. Very low removal of SMX by biodegradation, 22± 5% was found in an aerobic reactor in one study [31]. In the present MFC A/O system, the anoxic reactor can remove more of the target PPCPs because of the obvious growth of facultative bacteria within the MLSS and SPGRP biofilms. These bacterial populations were able to bring about removal rates for the PPCPs as follows: ACE (62.51%) > IBU (51.88%), both under anoxic condition. In one previous study, there was IBU biodegradation at 28%, with the concentration being 78 mg/L, which contrasted with the result for ACE at 11%, with the concentration being 66.12 mg/L, during anaerobic degradation at 37°C for 56 days [32].

### 4.2. Bacterial Species Involved in the Generation of Electricity by the MFC A/O System

Using a complex substrate like domestic wastewater that contains high strength PPCPs can help establish a diverse and electrochemically active microbial community using the MFC system. Some species in bacteria population that are able to produce electricity in a MFC were found to be abundant. Extracellular electron transfer was defined as electrons retrieved from the microbial oxidation of the organic substrates, namely, PPCP-containing sewage, in this study; these are then transferred to the anode.  Table 5 shows the specific bacterial species identified as being most closely related to the various MFC bacteria that have been identified in MFC systems. Anodophilic consortia, such as Geobacteraceae (identified as* Geobacter* spp. in this study), Clostridiaceae (identified as* Clostridium* spp. in this study, 10.24% clones), and various Proteobacteria species, have been shown to be able to generate a current in an anode chamber and are known to be able to transfer electrons to an electrode. For example, iron-reducing bacteria such as* Shewanella* and* Geobacter* spp. have been described as electrochemically active bacteria in MFC systems [33–35]. A* Leptothrix* sp. has been reported to be a type of Mn-oxidizing bacteria that bioaccumulates Mn oxides that can be used as cathodic reactants. The potential of a MFC that includes the reduction of Mn oxides deposited by* Leptothrix* spp. can be increased to about 300 mV and is able to deliver a current density up to two orders of magnitude higher than that reached using the reduction of O_2_ [36]. Rhodospirillales bacterium has been shown to be dominant in a cathodic MLSS rather than a biofilm; one possible reason for this is the fact that this is a light utilizing bacterial group capable of obtaining better illumination in suspension than as a biofilm [33].* Pelosinus* spp. are capable of fermenting lactate and coupling the oxidation of this compound to Fe^3+^; and such metal reduction in a microbial fuel cell can produce a maximum PD of 4.1 mW/m^2^ [34]. A* Treponema* sp. has been found to be present in a two-chambered PEM MFC that utilized active sludge enriched with chocolate industry wastewater [29]. A Lentisphaerae sp. has been found previously to be associated with the anode of a MFC system [37]. A* Pseudomonas* sp., a facultative anaerobic bacterium, is able to produce pyocyanin as a mediator and then uses these quorum signaling compounds to produce power [35]. A* Dechloromonas* sp. was identified as the most dominant species of anode bacteria in a butyrate-fed two-chamber MFC system [38].* Cupriavidus basilensis* has been shown to be involved in current production in a microbial fuel cell that used either acetate or phenol as a carbon source; in this case after 72 h in the MFC, 86% of the initial phenol concentration had been removed [39].

### 4.3. Bacterial Species Involved in the Biodegradation of PPCPs and Aromatic Compounds by the MFC A/O System

Table 6 outlines the specific bacterial species that are equipped with the ability to biodegrade aromatic compounds such as PPCPs, and these include bacteria associated with anaerobic biotransformation and aerobic ring cleavage, both of which were identified in the present study. Three species are known to have a direct relationship with PPCP biodegradation.* Dechloromonas* spp., which are *β*-Proteobacteria, have been detected in an A/O-MBR process that demonstrated good removal efficiency (88.5–99.5%) of antibiotics, including 500 *μ*g/L SMX, at various different HRTs [40].* Pseudomonas* spp. have been reported to biodegrade many pharmaceutical pollutants. For example, a high concentration of 2,000 mg/L ACE was able to be completely biodegraded as sole carbon source by a* Pseudomonas aeruginosa* isolated from the SBR treatment plant that processed ACE-contaminated wastewater [41].* Pseudomonas aeruginosa* is also able to biodegrade 1.3% of 6 mg/L SMX when this antibiotic is used as sole carbon source or 5.6% of 6 mg/L SMX when 0.5 g/L glucose is present as an additive [42]. In addition, a* Sphingomonas* sp. strain Ibu-2, which was found in a wastewater treatment plant, was shown to be able to biodegrade 500 mg/L IBU as sole carbon and energy source over 80 hrs [43]. Bacterial communities seem to have adapted to IBU biodegradation best under anoxic conditions. In such circumstances the biological degradation rate constant for IBU with time was found to increase from 16% at the beginning to 75% after 350 days.

The chemical structure of the biological metabolic products derived from PPCPs consists largely of benzene-ring compounds. Anaerobic benzene biodegradation by* Geobacter* sp. has been shown to occur in a petroleum-contaminated aquifer [44]. A* Hydrogenophaga* sp., which is a member of a heterogeneous aerobic benzene-degrading bacterial group, was found during the biological treatment in BTEX groundwater [45]. An overall 95% biodegradation of the lignin-related aromatic compound ferulic acid has been reported to occur with a* Cupriavidus* sp. when ferulic acid is used as a sole carbon and energy source [46]. A* Zoogloea* sp. has been shown to be able to biodegrade 98.6% of lubricating oil over 12 days with a HRT of 6 h. and an inflow rate of 33 L/h [47]. An Acidobacteria bacterium was found to be the dominant bacterial group during PAH bioremediation (3–5 aromatic rings) in soil and was also shown to be able to degrade benzene contaminated groundwater [48, 49]. A* Staphylococcus* sp., when immobilized on vermiculite, was used to remove hydrocarbons; this system used a fluidized bed bioreactor and synthetic water polluted with benzene, toluene, or naphthalene as sole sources of carbon and energy [50]. A Sphingobacteriales bacterium has been identified as part of an ethylbenzene-degrading sulfate-reducing consortium [51]. A* Prolixibacter* sp. has been identified by microbial enrichment to be able to biodegrade chlorinated pesticides that are present in contaminated sites of different geographical habitats of India [52]. A Burkholderiales bacterium has been identified as being able to degrade methyl tert-butyl ether (MTBE), a benzene, toluene, ethylbenzene, and xylene (BTEX) mixture, and tert-butyl alcohol (TBA) [53].

## 5. Conclusions

The pilot-scale MFC A/O sewage treatment was easily equipped with SPGRPs in order to treat municipal wastewater and to generate electricity in parallel with the biodegradation. The biological treatment of the PPCP-contained sewage demonstrated good performance over the time course of the experiment. A high removal efficiency of the target PPCPs was obtained after biofilms had formed on large specific surface areas available within the MFC A/O system. The ability to generate electricity using the SPGRP MFC is better than previous dual-chamber graphite MFC systems. A total of twenty bacterial species were identified as forming part of the MLSS and SPGRP biofilms and these identifications were used to clarify the possible functions of these microorganisms. These functions included both electrical generation and PPCP biodegradation. Practically, a scale-up of this SPGRP MFC A/O system for the treatment of real PPCP-contained sewage is needed and this should be applied to a commercial operation in the future. This will allow the design, operation, and maintenance of the system to be optimized. Importantly, such a system should be more efficient in terms of power use than conventional systems, without a significant increase in construction costs.

## Figures and Tables

**Table 1:** Components of the artificial PPCP-containing sewage (per liter) used in this study.

Component	Weight (mg)
SMX	2
ACE	30
IBU	20
Whole milk (KLIM, Nestlé)	119
Saccharide	30
Urea	11.76
KH_2_PO_4_	6.3
NH_4_Cl	5.6
FeCl_3_	0.14
Acetic acid (99.7%)	52.64 *μ*L
NaOH (10 N)	Drops were used to adjust the pH to 7.4

**Table 2:** Operating parameters for the MFC A/O reactor with a HRT of 8 h (mean ± SD)^2^.

Water parameters (unit)	Anoxic reactor (anode chamber)	Aerobic reactor (cathode chamber)
pH	8.07 ± 0.33	7.48 ± 0.31
Temperature (°C)	26.75 ± 1.23	29.58 ± 1.62
ORP (mV)	−393.51 ± 61.9	121.97 ± 42.61
DO (mg L^−1^)	ND^1^	4.22 ± 0.45
MLSS (mg L^−1^)	ND^1^	1956.07 ± 566.51
SVI (mL g^−1^)	ND^1^	218.82 ± 78.15
F/M	ND^1^	0.19 ± 0.1
F/V g BOD (m^3^ *·*day)^−1^	ND^1^	0.26 ± 0.14

^1^NA: not available.

^2^Average concentrations in the MFC A/O system during Phase I and Phase II (125 days).

**Table 3:** Changes in the sewage nutrients across the MFC A/O system (mean ± SD)^1^.

Water parameters (mg/L)	Influent	Anoxic reactor (anode)	Aerobic reactor (cathode)	Effluent
NH_4_^+^-N	1.767 ± 0.894	9.021 ± 3.623	0.087 ± 0.078	0.036 ± 0.049
NO_2_^−^-N	0.375 ± 0.152	0.258 ± 0.043	0.408 ± 0.211	0.344 ± 0.088
NO_3_^−^-N	1.555 ± 0.501	0.260 ± 0.076	0.335 ± 0.124	1.033 ± 0.670
PO_4_^3−^-P	1.318 ± 0.293	1.777 ± 0.497	0.501 ± 0.201	1.090 ± 0.422

^1^Average concentrations in the MFC A/O system during Phase I and Phase II (125 days).

**Table 4:** The richness index (RI) on the 109th day of Phase II obtained from the DGGE profiles allows assessments of the variation in biodiversity across the various areas of the novel MFC A/O system.

Same bands^1^/different bands^2^ (difference ratio^3^)		Anoxic reactor (anode)		
		MLSS	SPGRP biofilm	PEM biofilm
Aerobic reactor (cathode)	MLSS	3/18 (85.71%)	7/16 (69.56%)	7/13 (65.00%)
	SPGRP biofilm	7/15 (68.18%)	3/19 (86.36%)	4/16 (80.00%)
	PEM biofilm	5/13 (72.22%)	3/20 (86.96%)	3/18 (85.71%)

^1^Same bands are defined as the same location on the DGGE profile in Figure 3.

^2^Different bands are defined as the total number of different bands obtained when comparing each of two samples.

^3^The difference ratio is defined as the ratio of the number of different bands to all bands present.

**Table 5:** Bacteria identified by nucleic acid sequencing of 16S gene clones and by the searching of the GenBank database; these bacteria are associated with the generation of electricity by the MFC A/O system.

Accession number (closest match)	Sequence similarity	Species
KC502887	96%	Uncultured *Geobacter* sp.
FR774807	98%	Uncultured Clostridiales bacterium
FJ269104	96%	Iron-reducing bacterium
DQ234216	99%	Uncultured *Sulfurospirillum* bacterium
JF809001	100%	Uncultured *Leptothrix *sp.
JQ278984	99%	Uncultured Rhodospirillales bacterium
KC517355	87%	*Pelosinus* sp.
JQ086873	97%	Uncultured *Treponema* sp.
CU926806	97%	Uncultured *Lentisphaerae* sp.
KC871534	99%	*Pseudomonas *sp.
AF170354	99%	*Dechloromonas* sp.
HE662651	98%	*Cupriavidus basilensis *

**Table 6:** Bacteria identified by nucleic acid sequencing of 16S gene clones and by the searching of the GenBank database; these are associated with the biodegradation of PPCP and aromatic compounds in the MFC A/O system.

Accession no (Closest match)	Sequences similarity	Species
KC871534	99%	*Pseudomonas* sp.
AJ620198	99%	*Sphingomonas* sp.
AF170354	99%	*Dechloromonas* sp.
KC871534	96%	Uncultured* Geobacter *sp.
AB636293	97%	Uncultured* Hydrogenophaga *sp.
HE662651	98%	*Cupriavidus* sp.
HQ184339	98%	Uncultured* Zoogloea *sp.
JQ795417	96%	Uncultured Acidobacteria bacterium
KC310815	99%	*Staphylococcus* sp.
JQ723636	96%	Uncultured Sphingobacteriales bacterium
JN540151	95%	Uncultured* Prolixibacter *sp.
JF808996	99%	Uncultured Burkholderiaies bacterium
